# Knockdown of SHMT2 enhances the sensitivity of gastric cancer cells to radiotherapy through the Wnt/β-catenin pathway

**DOI:** 10.1515/biol-2022-0480

**Published:** 2022-09-19

**Authors:** Yu Mao, Tiyong Zhang

**Affiliations:** Department of General Surgery Unit 1, Yuhuan People’s Hospital, Taizhou, Zhejiang Province, 317699, China; Department of Radiology, Qianjiang Central Hospital of Chongqing Municipality, No. 63, West Ninth Road, Qianjiang District City, Chongqing, 409000, China

**Keywords:** gastric cancer, SHMT2, apoptosis, radiosensitivity, Wnt/β-catenin pathway

## Abstract

Gastric cancer (GC) is one of the most common malignant tumors. The mechanism of GC radioresistance and new radiosensitizers must be revealed and developed to treat GC. Serine hydroxymethyltransferase 2 (SHMT2) is responsible for encoding the mitochondrial form of the pyridoxal phosphate-dependent enzyme. SHMT2 plays a critical role in several types of cancers, while its possible effect on the radiological resistance in GC is still unclear. In this study, we investigated the role of SHMT2 in the radiological resistance of GC. Our data confirmed that SHMT2 was highly expressed in radiation-resistant GC cells. SHMT2 reduced the radiosensitivity of GC cells. In addition, SHMT2 is involved in radiation-induced GC cell apoptosis. Further, SHMT2 regulated the Wnt/β-catenin pathway, therefore reducing the radiosensitivity of GC cells *in vivo*. In conclusion, we revealed that depletion of SHMT2 enhanced the sensitivity of GC cells to interventional radiotherapy through the Wnt/β-catenin pathway.

## 1 Introduction

Gastric cancer (GC) is one of the most common malignant tumors [1,2]. Radiotherapy is the main treatment method for advanced GC patients. However, GC patients have limited benefits from radiation therapy, and most patients develop a tolerance to radiation therapy [3]. Although multiple genes are involved in mediating radiosensitivity in different cancers, the precise mechanisms of radioresistance in GC remain unknown.

Serine hydroxymethyltransferase 2 (SHMT2) is responsible for encoding the mitochondrial form of the pyridoxal phosphate-dependent enzyme [4]. SHMT2 is mainly involved in catalyzing the conversion of serine to glycine and the transfer of β-carbon from serine to hydrolyzed tetrahydrofluoric (THF) acid to form 5, 10-methylene tetrahydrofolate (Me THF) [5]. SHMT2 plays a regulatory role in cell proliferation [4]. High expression of SHMT2 has also been shown to predict the poor prognosis of GC and promote its progression [6]. SHMT2 levels are associated with elevated levels of β-catenin and CRC progression and can predict poor patient survival. Low expression of SHMT2 enhances sensitivity to 5-fluorouracil in colorectal cancer [7]. Higher SHTM2 expression was also observed in lapatinib-resistant breast cancer cells, and activation of SHMT2 enhanced breast cancer cell resistance to lapatinib [8].

Wnt signaling has been shown to play important roles in different cellular processes, including the regulation of cell proliferation, differentiation, migration, survival, and stem cell self-renewal [9]. Wnt inhibits β-catenin degradation, stabilizes β-catenin, and leads to β-catenin accumulation in the nucleus, ultimately controlling the expression of downstream target genes [10]. Studies have shown that the dysregulation of the Wnt/β-catenin signaling pathway is closely related to the occurrence of various human cancers [11]. In addition, the Wnt/β-catenin signaling pathway was found to mediate cancer radioresistance by participating in DNA damage repair. Together, these data support that activation of Wnt/β-catenin signaling plays a key role in tumorigenesis and radiological resistance.

In this study, we investigated the role of SHMT2 in the radioresistance of GC cells. Our data confirmed that SHMT2 affected the sensitivity of GC cells to interventional radiotherapy via the Wnt/β-catenin axis. Therefore, we confirmed that SHMT2 could serve as a target for the radioresistance of GC.

## 2 Materials and methods

### 2.1 Cell culture

The human GC cell lines, AGS and MKN45, were obtained from the ATCC (USA). To establish the radioresistant (RR) GC cell line, cells were exposed to a sublethal dose of irradiation for 6 months via the Small Animal Radiation Research Platform (Xstrahl, USA, 2018). The total exposure dose was 60 Gy. AGS and MKN45 were incubated with RPMI-1640 (HyClone, USA) supplied with 10% fetal bovine serum (Gibco, USA), as well as 1% penicillin–streptomycin. The resistant AGS and MKN45 cells were marked as AGS-R and MKN45-R, respectively.

### 2.2 Cell transfection

For the knockdown of SHMT2, a lentivirus-mediated delivery system, pLKD-CMV-G&PR-U6-shRNA (SHMT2) (4912, OBiO Technology, Shanghai, China), was used. Lentiviral shRNA targeting SHMT2 in this study was listed: 5′-CCGGAGAGTTGTGGACTTT-3′. Cell selection was performed with 2 μg/mL puromycin for 2 weeks.

### 2.3 Colony formation assay

Cells after indicated irradiation dose were seeded in 6-well plates (600/well). Ten days after irradiation, cells were fixed with 4% paraformaldehyde and cultured with 0.5% crystal violet. The colony numbers were counted manually.

### 2.4 Western blotting

Cell lysates were collected using RIPA buffer. Proteins were subjected to 10% sodium dodecyl sulfate-polyacrylamide gel electrophoresis (SDS-PAGE) and transferred onto polyvinylidene difluoride membranes. Then, membranes were incubated with 5% BSA followed by primary antibodies targeting SHMT2 (1:1,000, CST), Bcl-2 (1:1,000, Abcam, Cambridge, UK), Bax (1:1,000, Abcam, Cambridge, UK), β-catenin (1:1,000, CST), Axin (1:1,000, CST), c-myc (1:1,000, Abcam), and GAPDH (1:10,000; Abcam). Membranes were incubated with horse radish peroxide-conjugated secondary antibodies (The catalog number was: mice [cat. no. 7076] and rabbit [cat. no. 7074; both from Cell Signaling Technology, Inc.]) at a 1:5,000 for 2 h after rinsing in TBST for 15 min. The signals were detected with an ECL detection kit.

### 2.5 Flow cytometry

Cells were placed into a 24-well plate. After indicated treatment, 10^6^ cells were fixed and stained with fluorescein isothiocynate (FITC) and propidium iodide for 30 min in dark. Then, stained cells were washed by centrifugation and resuspend and analyzed on a BD FACS Canto II (BD Biosciences).

### 2.6 Statistical analysis

Data were represented by mean ± SD. Statistical analysis was performed using GraphPad. *P* < 0.05 was considered statistically significant. In the cell viability, colony formation, FCM, and Immunoblot assays, AGS-R vs AGS,^*^ *P* < 0.05 ^**^ *P* < 0.01, ***P < 0.001; ^^^ *P* < 0.05, ^^^^ *P* < 0.01, ^^^^^ *P* < 0.001 MKN45-R vs MKN45.

## 3 Results

### 3.1 Construction of RR GC cell line

**Figure 1:**
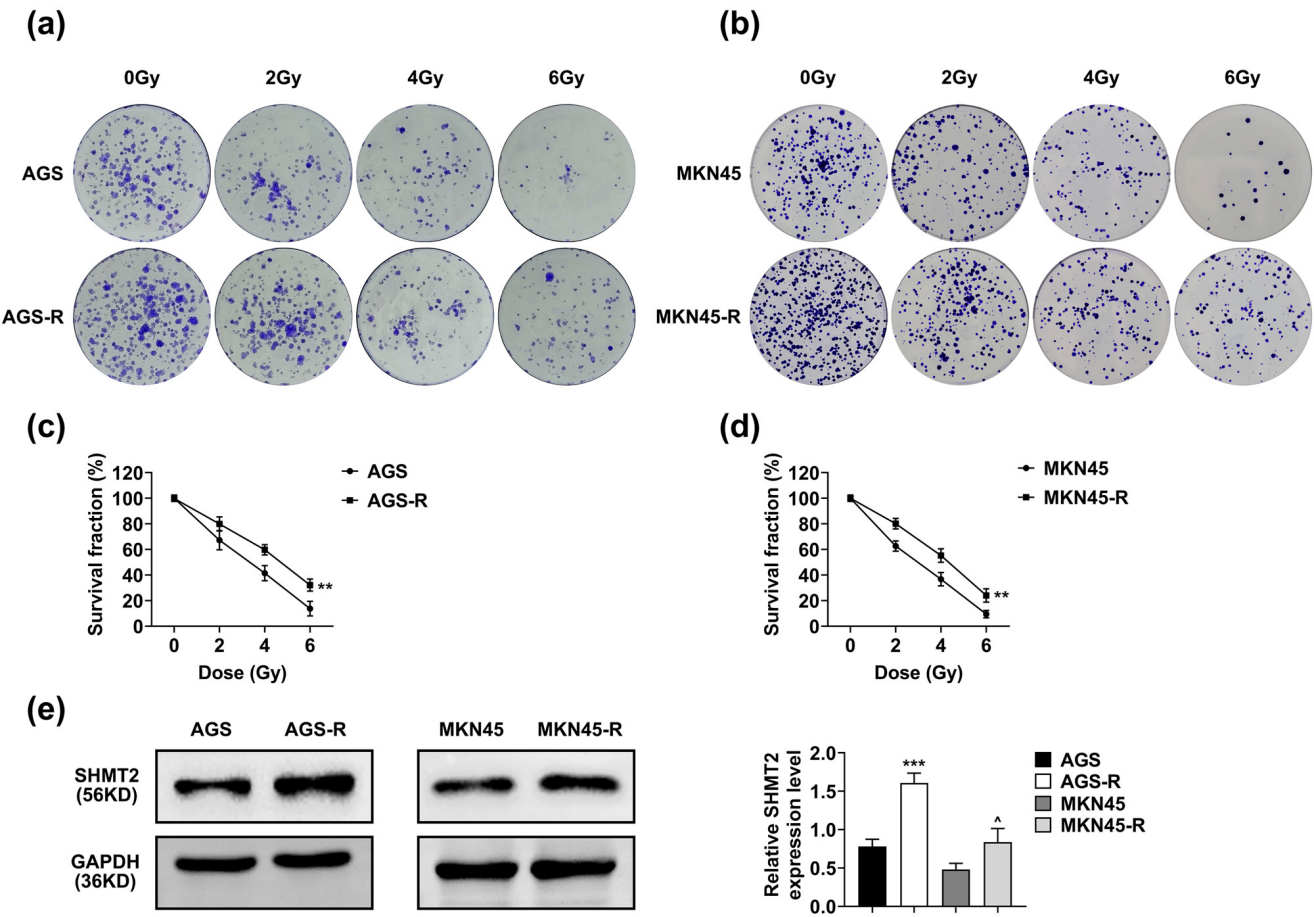
Construction of radioresistant (RR) GC cell line. (a) Colony formation assay in AGS and AGS-R cells treated with varying irradiations. The colony numbers were calculated. (b) Colony formation assay in MKN45 and MKN45-R cells treated with varying irradiations. The colony numbers were calculated. (c and d) The survival fraction as quantified from (a) and (b). (e) The expression of SHMT2 in AGS, AGS-R, MKN45, and MKN45-R cells. Data were represented by mean ± SD. ^**^ *P* < 0.01, ***P < 0.001 AGS-R vs AGS, and ^^^ *P* < 0.05 MKN45-R vs MKN45.

For the construction of the RR GC cell line, AGS and MKN45 cells were exposed to fractioned irradiation at a total dose of 60 Gy for 6 months and validated to be RR. Colony number was significantly higher in the RR cells when compared with parental at varying doses of irradiation (Figure 1a and b). The quantification of survival fraction was also higher in RR cells (Figure 1c and d). Moreover, SHMT2 was upregulated in the RR of GC cells (Figure 1e). These results suggested that SHMT2 was upregulated in RR cells.

### 3.2 SHMT2 ablation sensitizes RR GC to irradiation

**Figure 2:**
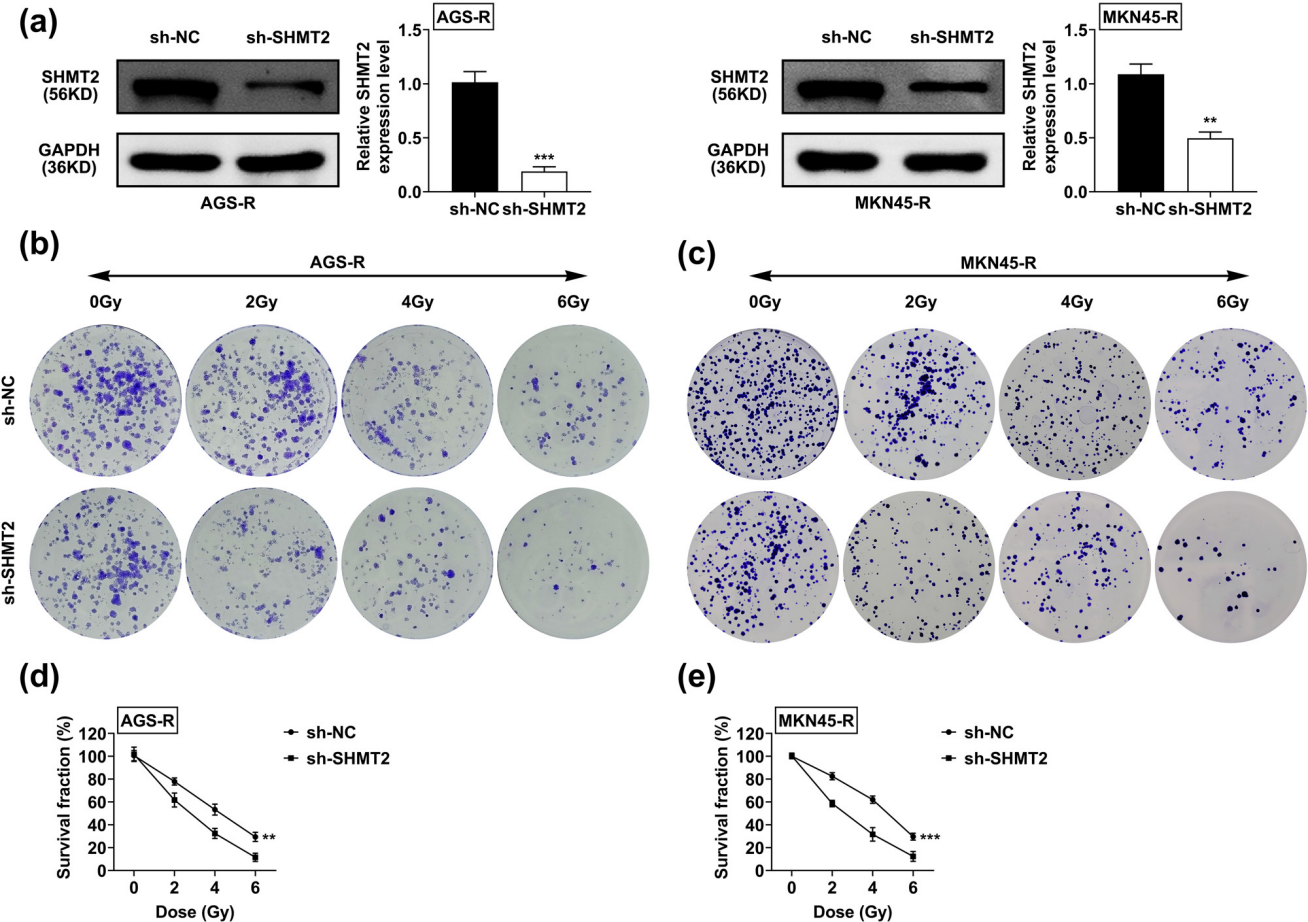
SHMT2 ablation sensitizes RR GC to irradiation. (a) The immunoblot confirmed the silencing efficiency in AGS-R and MKN45-R cells. (b) Colony formation assay in AGS-R cells treated with sh-NC and sh-SHMT2 and varying irradiations. (c) Colony formation assay in MKN45-R cells treated with sh-NC and sh-SHMT2 and varying irradiations. (d and e) The survival fraction as quantified from A, B. Data was represented by mean ± SD. ^**^ *P* < 0.01, ***P < 0.001.

To reveal the role of SHMT2 in GC sensitivity to irradiation, we knocked down SHMT2 in GC cells and the knockdown efficiency was confirmed (Figure 2a). The role of SHMT2 in irradiation sensitivity was determined by colony formation assay. Interestingly, downregulation of SHMT2 decreased cell colony formation in response to an increased dose of irradiation compared with RR GC cells (Figure 2b and c). The decreased survival fraction in SHMT2 knockdown cells also confirmed the increased sensitivity to irradiation in RR cells (Figure 2d and e).

### 3.3 SHMT2 knockdown promoted radiation-induced GC cell apoptosis

**Figure 3:**
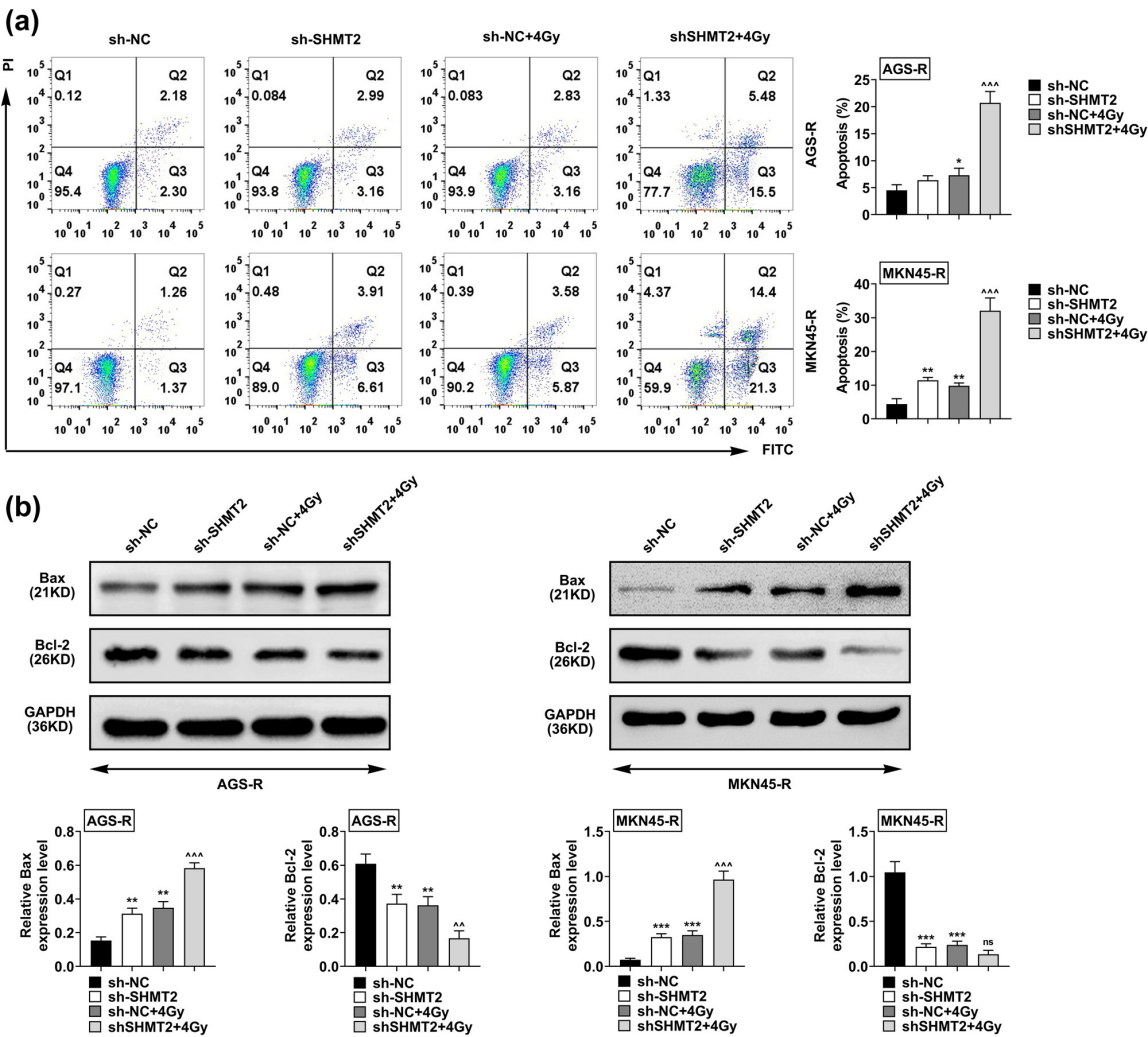
SHMT2 promoted radiation-induced GC cell apoptosis. (a) Cell apoptosis in AGS-R and MKN45-R cells treated with sh-NC and sh-SHMT2 and irradiations. (b) The level of bax and bcl-2 in AGS-R and MKN45-R cells treated with sh-NC and sh-SHMT2 and irradiations. The sh-NC was used as the statistical control group in the whole experiment. Data were represented by mean ± SD. ^**^ *P* < 0.01, ***P < 0.001 AGS-R vs AGS, ^^^^ *P* < 0.01, and ^^^^^ *P* < 0.001 MKN45-R vs MKN45.

To detect the effect of SHMT2 on cell apoptosis, flow cytometry was performed in RR GC cells. Flow cytometry analysis showed that SHMT2 knockdown led to enhanced cell apoptosis in 4 Gy irradiation conditions (Figure 3a). Moreover, the level of Bax was increased and Bcl-2 was repressed in SHMT2 knockdown RR cells with and without irradiation (Figure 3b). Overexpression of SHMT2 promoted viability and suppressed the apoptosis of RR GC cells (Figure S1a–c). Taken together, SHMT2 knockdown induces cell apoptosis of GC stimulated with radiation.

### 3.4 SHMT2 affected the sensitivity of GC cells to interventional radiotherapy via the Wnt/β-catenin pathway

**Figure 4:**
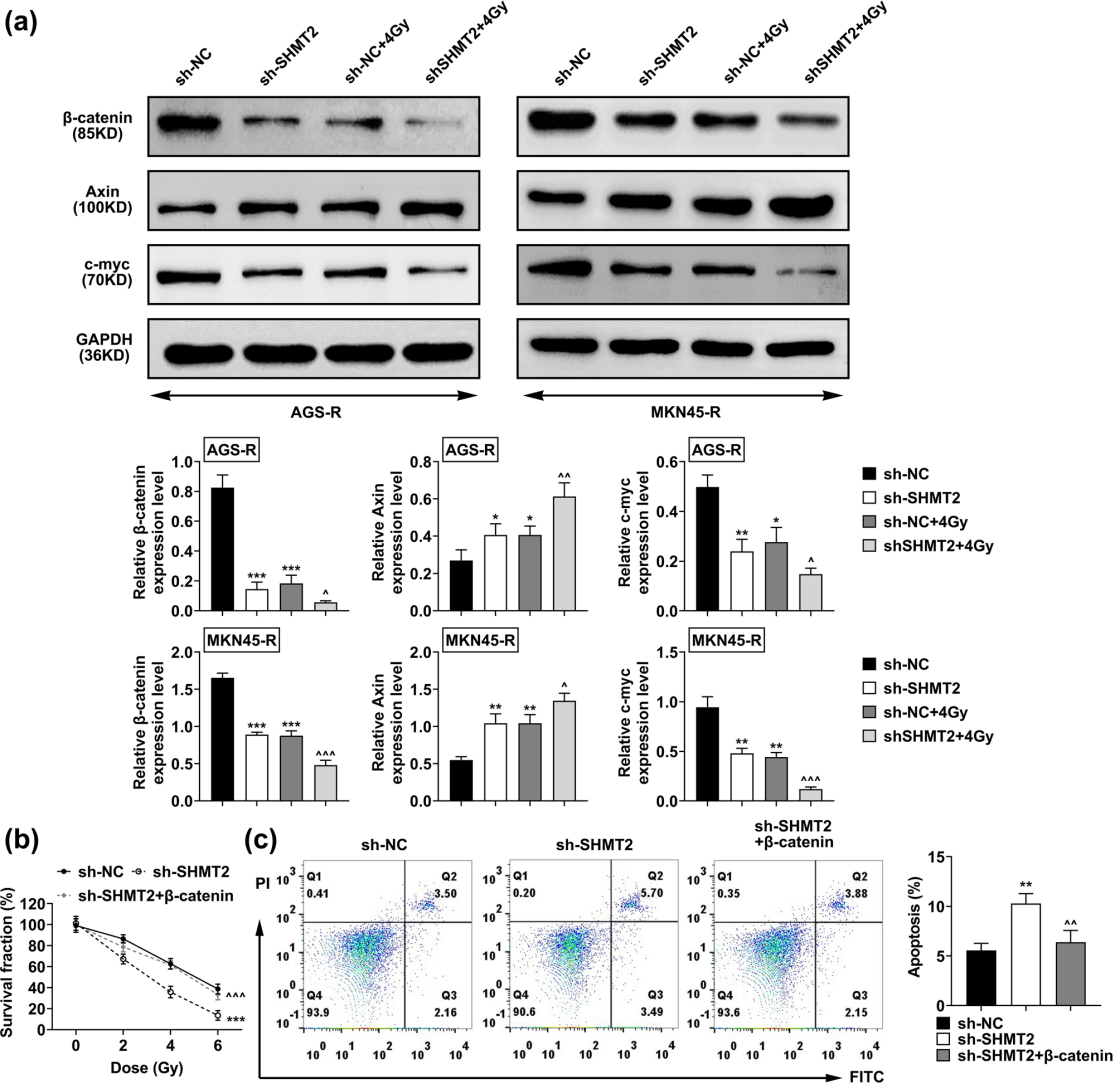
SHMT2 affected the sensitivity of GC cells to interventional radiotherapy via the Wnt/β-catenin pathway. (a) The expression of β-catenin, axin, and c-myc in AGS-R and MKN45-R cells treated with sh-NC and sh-SHMT2 and irradiations. (b) CCK-8 assays showed the viability of MKN45-R cells upon the indicated transfection. (c) FCM assays showed the apoptosis levels of MKN45-R cells upon the indicated transfection. The sh-NC was used as the statistical control group in the whole experiment. Data were represented by mean ± SD.^**^ *P* < 0.01, ***P < 0.001 AGS-R vs AGS, ^^^ *P* < 0.05, and ^^^^^ *P* < 0.001 MKN45-R vs MKN45.

We noticed a reduced level of β-catenin and c-myc, and an enhanced level of axin in SHMT2 knockdown RR cells with or without irradiation (Figure 4a). We further performed rescue assays through CCK-8 and FCM assays in MKN45-R cells. Our results revealed that the knockdown of SHMT2 suppressed the viability and stimulated apoptosis (Figure 4b and c), whereas overexpression of β-catenin reversed these effects in MKN45-R cells (Figure 4b and c). Altogether, knockdown of SHMT2 enhanced the sensitivity of GC cells to interventional radiotherapy through the Wnt/β-catenin pathway.

## 4 Discussion

GC is a malignant tumor with high morbidity and mortality, which is difficult to eradicate except for surgical resection after early detection [12]. Existing treatment methods, such as surgery, radiotherapy, and chemotherapy, are limited for GC treatment [13]. In the past, radiotherapy was not suitable for the treatment of GC either alone or in combination [14]. The main reasons include the following: GC is mostly adenocarcinoma, and adenocarcinoma is not sensitive to radiation [15]. Here, we noticed downregulation of SHMT2 enhanced the sensitivity of GC cells to interventional radiotherapy.

We constructed a radiotherapy-resistance GC cell line and performed a series of *in vitro* assays such as Immunoblot, CCK-8, and FCM, and the results revealed that SHMT2 was highly expressed in radiotherapy-resistance GC cells and reduced the radiosensitivity of GC cells, and stimulate cell apoptosis. In fact, proliferation, and apoptosis could be affected by radiotherapy in cancer cells. In RR cancer cells, radiotherapy had no obvious effects on the proliferation and apoptosis of cancer cells. The effects of SHMT2 on cancer progression and metastasis have been widely revealed. For example, SHMT2 knockdown disrupted the TCF3 transcriptional survival program in lymphoma [16]. Overexpression of SHMT2 was correlated with a poor prognosis and promoted tumor growth in bladder cancer and thyroid cancer [17,18]. In addition, loss of SHMT2 could mediate 5-fluorouracil chemoresistance in colorectal cancer via promoting autophagy [19]. Similarly, we revealed its effects on the radiotherapy sensitivity of GC cells. The depletion of SHMT2 could suppress the progression of tongue squamous cell carcinoma by mediating the cell cycle [20]. We should further detect the effects of SHMT2 on the cell cycle of RR cancer cells. Another study indicated that higher SHTM2 expression was also observed in lapatinib-resistant breast cancer cells, and activation of SHMT2 enhanced breast cancer cell resistance to lapatinib [21]. All these studies confirmed that SHMT2 could serve as a promising target for cancer treatment.

Notably, SHMT2 could drive the progression and metastasis of colorectal cancer via suppressing β-catenin degradation. Similarly, here, we also showed that SHMT2 affected the sensitivity of GC cells to interventional radiotherapy via this pathway. This pathway has been revealed to affect the proliferation and motility of tumors and affect the EMT progression of several types of cancers, including GC [9]. Multiple proteins or drugs affected GC progression via this pathway. Importantly, this pathway was involved in the radiotherapy resistance of GC cells. For example, DOCK6 contributed to chemo- and radioresistance of GC cells via this pathway [10]. LincRNA-p21 promoted the sensitivity of radiotherapy for GC via this pathway [22]. These studies confirmed that Wnt/β-catenin was a critical target to improve the radiotherapy-resistance of GC cells.

In summary, we constructed a RR GC cell line and investigated the role of SHMT2 in this cell line. Our data confirmed that SHMT2 was highly expressed in RR GC cells and affected the sensitivity of GC cells to radiotherapy via the Wnt/β-catenin pathway. We therefore confirmed that SHMT2 could serve as a target for the radioresistance of GC.

## Supplementary Material

Supplementary Figure

## References

[1] Abnet CC, Freedman ND, Hu N, Wang Z, Yu K, Shu XO, et al. A shared susceptibility locus in PLCE1 at 10q23 for gastric adenocarcinoma and esophageal squamous cell carcinoma. Nat Genet. 2010;42(9):764–7. 10.1038/ng.649 PMC2947317 20729852

[2] Camilloni A, Nati G, Maggiolini P, Romanelli A, Latina R. Chronic non-cancer pain in primary care: an Italian cross-sectional study. Signa Vitae. 2021;7(2):54–62.

[3] Luo D, Gao Y, Wang S, Wang M, Wu D, Wang W, et al. Genetic variation in PLCE1 is associated with gastric cancer survival in a Chinese population. J Gastroenterol. 2011;46(11):1260–6. 10.1007/s00535-011-0445-3 21837401

[4] Liu C, Wang L, Liu X, Tan Y, Tao L, Xiao Y, et al. Cytoplasmic SHMT2 drives the progression and metastasis of colorectal cancer by inhibiting beta-catenin degradation. Theranostics. 2021;11(6):2966–86. 10.7150/thno.48699 PMC7806468 33456583

[5] Wilke AC, Doebele C, Zindel A, Lee KS, Rieke SA, Ceribelli M, et al. SHMT2 inhibition disrupts the TCF3 transcriptional survival program in Burkitt lymphoma. Blood. 2020;139(4). 10.1182/blood.2021012081. PMC8938936 34624079

[6] Li J, Zhang B, Gan M, Li Y, He L, Yue W, et al. Generation of SHMT2 knockout human embryonic stem cell line (WAe009-A-67) using CRISPR/Cas9 technique. Stem Cell Res. 2021;57:102581. 10.1016/j.scr.2021.102581 34688993

[7] Tong J, Krieger JR, Taylor P, Bagshaw R, Kang J, Jeedigunta S, et al. Cancer proteome and metabolite changes linked to SHMT2. PLoS One. 2020;15(9):e0237981. 10.1371/journal.pone.0237981 PMC7480864 32903271

[8] Zeng Y, Zhang J, Xu M, Chen F, Zi R, Yue J, et al. Roles of mitochondrial serine hydroxymethyltransferase 2 (SHMT2) in human carcinogenesis. J Cancer. 2021;12(19):5888–94. 10.7150/jca.60170 PMC8408114 34476002

[9] Garcia-Cazorla A, Verdura E, Julia-Palacios N, Anderson EN, Goicoechea L, Planas-Serra L, et al. Impairment of the mitochondrial one-carbon metabolism enzyme SHMT2 causes a novel brain and heart developmental syndrome. Acta Neuropathol. 2020;140(6):971–5. 10.1007/s00401-020-02223-w PMC7665968 33015733

[10] Escande-Beillard N, Loh A, Saleem SN, Kanata K, Hashimoto Y, Altunoglu U, et al. Loss of PYCR2 causes neurodegeneration by increasing cerebral glycine levels via SHMT2. Neuron. 2020;107(1):82–94 e6. 10.1016/j.neuron.2020.03.028 32330411

[11] Tramonti A, Cuyas E, Encinar JA, Pietzke M, Paone A, Verdura S, et al. Metformin Is a Pyridoxal-5′-phosphate (PLP)-Competitive Inhibitor of SHMT2. Cancers. 2021;13(16):4009. 10.3390/cancers13164009 PMC8393646 34439169

[12] Tanaka K, Arao T, Maegawa M, Matsumoto K, Kaneda H, Kudo K, et al. SRPX2 is overexpressed in gastric cancer and promotes cellular migration and adhesion. Int J Cancer. 2009;124(5):1072–80. 10.1002/ijc.24065 19065654

[13] Zhang X, Yashiro M, Ren J, Hirakawa K. Histone deacetylase inhibitor, trichostatin A, increases the chemosensitivity of anticancer drugs in gastric cancer cell lines. Oncol Rep. 2006;16(3):563–8. 16865256

[14] Liu J, Geng Z, Zhang Y, Alharbi SA, Shi Y. Sesquiterpenoid bilobalide inhibits gastric carcinoma cell growth and induces apoptosis both in vitro and in vivo models. J Biochem Mol Toxicol. 2021;35(5):e22723. 10.1002/jbt.22723 33511709

[15] Yang Z, Jiang X, Zhang Z, Zhao Z, Xing W, Liu Y, et al. HDAC3-dependent transcriptional repression of FOXA2 regulates FTO/m6A/MYC signaling to contribute to the development of gastric cancer. Cancer Gene Ther. 2021;28(1–2):141–55. 10.1038/s41417-020-0193-8 32655129

[16] Hashizume O, Ohnishi S, Mito T, Shimizu A, Ishikawa K, Nakada K, et al. Epigenetic regulation of the nuclear-coded GCAT and SHMT2 genes confers human age-associated mitochondrial respiration defects. Sci Rep. 2015;5:10434. 10.1038/srep10434 PMC5377050 26000717

[17] Zhang P, Yang Q. Overexpression of SHMT2 predicts a poor prognosis and promotes tumor cell growth in bladder cancer. Front Genet. 2021;12:682856. 10.3389/fgene.2021.682856 PMC8212063 34149818

[18] Jin M, Lee WK, You MH, Jang A, Cheng SY, Kim WG, et al. SHMT2 expression as a diagnostic and prognostic marker for thyroid cancer. Endocr Connect. 2021;10(6):630–6. 10.1530/EC-21-0135 PMC8240706 34010151

[19] Cao J, Sun L, Aramsangtienchai P, Spiegelman NA, Zhang X, Huang W, et al. HDAC11 regulates type I interferon signaling through defatty-acylation of SHMT2. Proc Natl Acad Sci U S Am. 2019;116(12):5487–92. 10.1073/pnas.1815365116 PMC6431144 30819897

[20] Bernhardt S, Bayerlova M, Vetter M, Wachter A, Mitra D, Hanf V, et al. Proteomic profiling of breast cancer metabolism identifies SHMT2 and ASCT2 as prognostic factors. Breast Cancer Res. 2017;19(1):112. 10.1186/s13058-017-0905-7 PMC5637318 29020998

[21] Luo L, Zheng Y, Lin Z, Li X, Li X, Li M, et al. Identification of SHMT2 as a potential prognostic biomarker and correlating with immune infiltrates in lung adenocarcinoma. J Immunol Res. 2021;2021:6647122. 10.1155/2021/6647122 PMC8049788 33928169

[22] Cuthbertson CR, Arabzada Z, Bankhead A 3rd, Kyani A, Neamati N. A review of small-molecule inhibitors of one-carbon enzymes: SHMT2 and MTHFD2 in the spotlight. ACS Pharmacol Transl Sci. 2021;4(2):624–46. 10.1021/acsptsci.0c00223 PMC8033767 33860190

