# Supplementary material for: Knockdown of SHMT2 enhances the sensitivity of gastric cancer cells to radiotherapy through the Wnt/β-catenin pathway
Source: Open Life Sci. 2022 Sep 19;17(1):1249–55. doi: 10.1515/biol-2022-0480 (PMC9490860; doi:10.1515/biol-2022-0480)
Supplement: Supplementary Figure [file biol-2022-0480-sm.pdf]

Supplementary materials

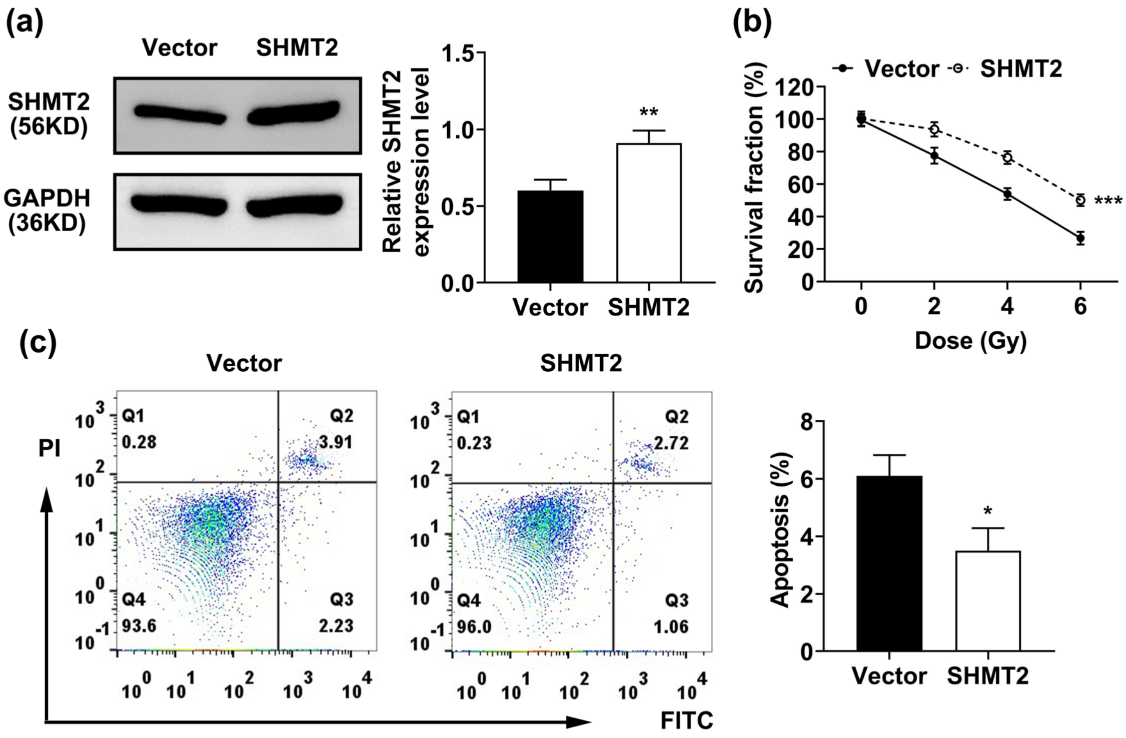

Figure S1: Effects of SHMT2 overexpression in MKN45-R cells. (a) The expression of SHMT2 in MKN45-R cells transfected with control and SHMT2 plasmids. (b) CCK-8 assays showed the viability of MKN45-R cells upon the indicated transfection. (c) FCM assays showed the apoptosis levels of MKN45-R cells upon the indicated transfection. Data was represented by mean ± SD. $^{*}P < 0.05$, $^{**}P < 0.01$, $^{***}P < 0.001$.
